# The relation between epidermal growth factor receptor mutations profiles and smoking patterns in patients with lung adenocarcinoma: A cross‐sectional study

**DOI:** 10.1002/hsr2.1369

**Published:** 2023-07-05

**Authors:** Seyedeh Yasamin Parvar, Alireza Rezvani, Rezvan Ghaderpanah, Mohammadhossein Hefzosseheh, Shakila Rafiei, Ahmad Monabati

**Affiliations:** ^1^ Student Research Committee Shiraz University of Medical Sciences Shiraz Iran; ^2^ Department of internal medicine, Autophagy Research Center Shiraz University of Medical Science Shiraz Iran; ^3^ Department of Pathology Shiraz University of Medical Sciences Shiraz Iran

**Keywords:** adenocarcinoma of the lung, cigarette smoking, EGFR gene, non‐small cell lung cancer

## Abstract

**Background:**

Non‐small cell lung cancer (NSCLC) accounts for 85% of lung cancer cases, with smoking being a critical risk factor. The identification of NSCLC patients harboring epidermal growth factor receptor (EGFR) mutations, sensitized to tyrosine kinase inhibitors, has revolutionized treatment plans, resulting in improved clinical responses and reduced chemotherapy toxicity. This study aimed to assess the relationship between EGFR mutations and smoking patterns in patients diagnosed with lung adenocarcinoma referred to major pathologic laboratories.

**Methods:**

This cross‐sectional study included 217 NSCLC patients aged above 18 years. Molecular abnormalities of the EGFR gene were analyzed by polymerase chain reaction amplification of exons 18–21 accompanied by Sanger sequencing. Then, the data were analyzed using the SPSS 26 software. Logistic regression analysis, *χ* ^2^ test, and Mann–Whitney *U* test were used to evaluate the relation between EGFR mutations and smoking patterns.

**Results:**

EGFR mutations were identified in 25.3% of patients, predominantly involving deletion in exon 19 (61.8%). For most of the mutant EGFR patients, the majority were nonsmokers (81.8%), and 52.7% were female patients. Besides, the median duration of smoking was 26 years and the median frequency of smoking was 23 pack‐years in the mutant EGFR group, both of which were lower compared to the wild mutant group. Moreover, female gender, current, and heavy smoking were significantly correlated with EGFR mutations based on the univariate logistic regression analysis (*p*: 0.004, 0.005, and 0.001, respectively).

**Conclusions:**

Female gender and nonsmoker status were strongly associated with positive EGFR mutations. While guidelines traditionally recommended EGFR testing primarily for female nonsmokers with advanced NSCLC, our study in line with the recently published evidence has shown a significant prevalence of positive EGFR mutations among male patients and smokers. Therefore, routine mutation testing is suggested for all NSCLC patients. Considering the limited access to EGFR testing laboratories in developing countries, the results of such epidemiological surveys can assist oncologists in choosing the most suitable treatment plan.

## 1 INTRODUCTION

Lung cancer (LC) is the leading cause of cancer deaths and the second most commonly diagnosed cancer worldwide. It is the primary cause of cancer‐related deaths in males and the second in females.^1^ According to recent global epidemiology data on LC that has been published recently, the highest frequency of LC was seen in Asian countries close to Eastern Europe such as Armenia, Turkey, and Kazakhstan. In contrast, Middle Eastern countries like Saudi Arabia and Yemen have reported the lowest frequency.^2^

LC has been associated with multiple risk factors, including genetic, environmental, and occupational factors, such as active and passive smoking, various genomic changes, pre‐existing lung diseases, and indoor and outdoor air pollution.^3^, ^4^, ^5^ The incidence of LC varies due to different cigarette smoking patterns.^2^ Studies conducted in our country have shown that smoking cigarettes is the most significant risk factor observed in approximately 60% of LC patients.^6^, ^7^ The related systematic review and meta‐analyses have also shown a strong association between water‐pipe smoking and the risk of LC progression.^8^

Non‐small cell lung cancer (NSCLC) is the most common type of LC, accounting for 85% of cases. NSCLC has two histological subtypes, that is, adenocarcinoma, which is the most prevalent histologic subtype in both sexes,^2^, ^6^ and squamous cell carcinoma. Additionally, approximately one‐third of NSCLC patients, especially those with adenocarcinoma, have been found to harbor epidermal growth factor receptor (EGFR) mutations.^9^, ^10^ EGFR mutations, the most common mutations observed in female and nonsmoker NSCLC patients,^5^ lead to increase growth signal within the cell, eventually resulting in the development of cancer cells.^11^ Recently, the recognition of NSCLC patients harboring EGFR mutations, who respond well to tyrosine kinase inhibitors (TKIs) like Gefitinib or Erlotinib, has revolutionized treatment plans, resulting in an improvement in clinical responses and quality of life. Therefore, first‐line treatment with targeted therapies has shown superior clinical outcomes compared to conventional cytotoxic chemotherapy regimens in patients with EGFR mutations.^12^, ^13^

There is limited information regarding the epidemiology of EGFR mutations and smoking patterns in our region, and most surveys have not evaluated detailed smoking profiles and habits in EGFR‐positive group. Hence, the current study aims to assess the relationship between EGFR mutations and smoking patterns among patients diagnosed with lung adenocarcinoma referred to pathological laboratories.

## 2 PATIENTS AND METHODS

### 2.1 Study design and participant recruitment

This cross‐sectional multicenter study was conducted in compliance with the Declaration of Helsinki and was approved by the university's Ethics Committee. The study period spanned from September 2017 to March 2022 and included all patients above 18 years of age who had histologically confirmed adenocarcinoma. The patients were referred to one of the three reference laboratories at the onset of diagnosis and agreed to participate. Patients who lacked a contact number, were not cooperative and informative, provided incomplete data sheets, were under the age of 18 (pediatric cases), had nonadenocarcinoma histology, and those with secondary LC, received previous chemotherapy, or had insufficient samples for EGFR testing were excluded from the study.

### 2.2 Sample size measurements

**Figure 1:**
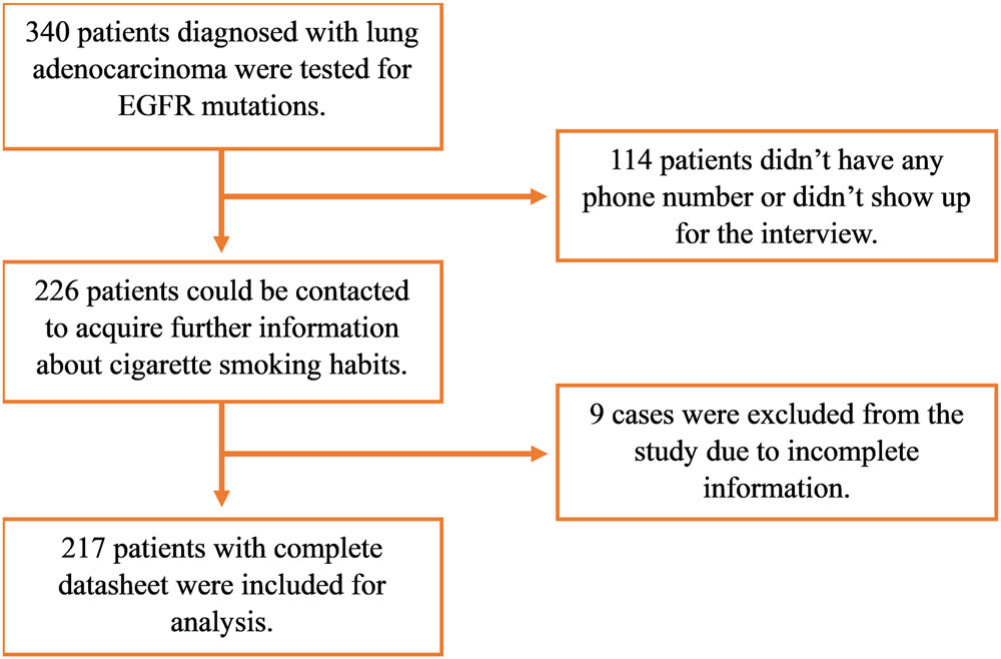
Flow diagram of participant recruitment in the study. EGFR, epidermal growth factor receptor.

On the basis of study by Wei et al.,^14^ considering the frequency of EGFR mutation as 21%, confidence interval (CI) of 95%, power of 21%, and *d* (margin of error) = 0.05, and using the *n* = *Z* ^2^ × *P*(1 − *P*)/*d* ^2^ formula, 255 samples were determined for the study. A flow diagram illustrating the participant recruitment process is provided in Figure 1.

### 2.3 Data collection and outcome measures

Demographic and pathological characteristics, including age, gender, EGFR mutation testing results, and phone number, were obtained from the laboratories. After a thorough explanation of the study's aim and obtaining consent to participate, the following information was collected over the phone from patients or they first‐degree relatives (in case of death at the time of interview): marital status, living status (alive or dead), cigarette smoking ha, frequency of smoking, duration of smoking, and duration of disease. Then, the data were entered and coded into the Microsoft Excel software 2016 (Version 2212).

Considering the cigarette smoking pattern, the patients were classified into three categories:

- Current smokers (still smoking or quitting smoking less than 6 months before the disease diagnosis).
- Former smokers (quitting smoking more than 6 months before the disease diagnosis).
- Never smokers.

The smoking frequency was determined by multiplying the number of cigarette packs smoked per day by the number of smoking years. Heavy smokers smoke greater than or equal to 20 or more cigarette packs a year, while light smokers are those who smoke less than 20 packs per year. The duration of the disease was measured from the onset of diagnosis by EGFR testing till the time of death or the time of the study.

### 2.4 EGFR mutation testing

Molecular abnormalities of the EGFR gene were assessed among all included patients and all tests were verified by two expert pathologists according to the World Health Organization's classification of lung tumors standards, updated in 2021.^15^ All 217 samples were collected as fresh resection specimens or paraffin embedded tissue sections of lung biopsy tumor blocks with the highest percentage of tumor cells (>10%). Polymerase chain reaction (PCR) amplification of exons 18–21 was done, followed by Sanger sequencing of the product. Although this test has high specificity, its sensitivity in detecting mutations is limited. The PCR amplification procedure was conducted in three stages within 2 h of execution. Primers used for sequencing the EGFR gene have been presented in Table 1. The first stage was a 5‐min denaturation at 95°C. The second stage involved 15 cycles of 25 s at 95°C, 20 s at 64°C, and 20 s at 72°C. The third stage included 31 cycles of 25 s at 93°C, 35 s at 60°C, and 20 s at 72°C. The required information was gathered at 60°C during stage three. In case of undetermined results, the “Easy® EGFR” Kit (Diatech Pharmacogenetics; Cat. No. RT003) contains eight oligonucleotide mixes that detect 30 mutations of the oncogene EGFR by real‐time PCR was used. Additionally, a specific oligo‐control mix enables the evaluation of the quality and quantity of the DNA in each sample. If the EGFR is wild in these hot points, other mutations will be investigated. The EGFR mutation‐positive samples were classified into five subtypes. The details of these subtypes have been presented in Supporting Information: Table 1. All EGFR mutation test results were classified and analyzed in three categories, namely wild type, deletion in exon 19, and others. It should be noted that the patients did not undergo chemotherapy or radiotherapy before their surgical resection. This approach was taken to prevent any up or downregulation of cell‐cycle proteins caused by DNA damage.

**Table 1:** Primers used for sequencing of the epidermal growth factor receptor (EGFR) gene.

No.	Oligonucleotide name	Sequence (5′–>3′)	Yield (nmol)	Yield (OD)	Yield (µg)
1.	EGFR18F	CATGGTGAGGGCTGAGGTGA (20)	45.1	10.38	283
2.	EGFR18R	CCCCACCAGACCATGAGAGG (20)	56.0	12.32	340
3.	EGFR19F	GTGCATCGCTGGTAACATCCA (21)	53.2	12.06	341
4.	EGFR19R	GGAGATGAGCAGGGTCTAGAGCA (23)	48.8	13.41	351
5.	EGFR20F	CGCATTCATGCGTCTTCACC (20)	52.2	10.26	313
6.	EGFR20R	CTATCCCAGGAGCGCAGACC (20)	48.6	10.39	295
7.	EGFR21F	TGGCATGAACATGACCCTGAA (21)	49.3	11.87	318
8.	EGFR21R	CAGCCTGGTCCCTGGTGTC (19)	55.6	10.24	321

Abbreviation: OD, optical density.

### 2.5 Statistical analysis

The data were presented as mean ± standard deviation (SD), median (interquartile range), and number (%). An Independent *t* test was employed to compare the EGFR‐positive and EGFR‐negative groups regarding age. A *χ* ^2^ test was used for categorical variables while the Mann–Whitney *U* test was utilized to evaluate the duration of disease, duration, and frequency of smoking which were nonparametric. Univariate and multivariate logistic regression analyses were conducted to identify predictors of EGFR mutations. The data were analyzed using the IBM Statistical Package for Social Sciences (SPSS, version 26; SPSS Inc.). *p* < 0.05 was considered statistically significant.

## 3 RESULTS

A total of 217 patients diagnosed with NSCLC were included in the study. EGFR mutations were identified in 25.3% of patients (*n*: 55), with the majority of these mutations being deletion in exon 19 (61.8%). The mean age at admission was 61 years (SD = 12.62), ranging from 19 to 91 years. The male‐to‐female ratio was 1.7 and the *χ* ^2^ test results indicated that male patients comprised a significantly higher proportion of wild‐type EGFR patients (69.1%, *p*: 0.006). Conversely, most mutant EGFR patients were female (52.7%). The median duration of disease was 12 years in our population and higher in the mutant group (*p* value according to the Mann–Whitney *U* test: 0.099). Additionally, 96.8% of the patients were married (*p*: 0.195), and 57.9% were dead at the time of the interview.

In terms of smoking status, nonsmokers accounted for 61.3% of all patients, while former smokers and current smokers each comprised 19.4% of the total. In addition, only four patients (7.3%) with mutant EGFR were current smokers, which was significant according to the *χ* ^2^ test (*p*: 0.001). Furthermore, the median duration and frequency of smoking were lower in the mutant group, which was not significant based on the Mann–Whitney *U* test. Moreover, heavy smokers were 2.4 times more frequent than light ones in the wild group, and most light‐ and heavy‐smoker patients presented with wild‐type EGFR (84.6% and 89.6%, respectively). Additionally, the patients who never smoked showed higher mutation rates than light and heavy smokers.

The patient's demographic characteristics and smoking status associated with the EGFR mutation status have been presented in Table 2.

**Table 2:** Demographic characteristics and smoking status of patients according to the EGFR mutation status (*n*: 217).

Variables	Total	Wild EGFR (*n* = 162)	Mutant EGFR (*n* = 55)	*p* Value*
Age^a^ (years) mean (±SD)	60.99 (±12.62)	60.96 (±12.35)	61.07 (±13.51)	0.953
Gender^b^ *N* (%)				0.006
Male	138 (63.6)	112 (69.1)	26 (47.3)	
Female	79 (36.4)	50 (30.9)	29 (52.7)	
Marital status^b^ *N* (%)				0.195
Single	7 (3.2)	7 (4.3)	0 (0)	
Married	210 (96.8)	155 (95.7)	55 (100)	
Duration of disease^c^ (years) median [IQR] (*n* = 191)	12.0 [7.0–19.0]	11.0 [6.0–18.0]	16.0 [8.0–20.0]	0.099
Living status^b^ *N* (%) (*n* = 190)				1.000
Alive	80 (42.1)	60 (41.9)	20 (42.6)	
Death	110 (57.9)	83 (58.0)	27 (57.4)	
Smoking status^b^ *N* (%)				0.001
Nonsmoker	133 (61.3)	88 (54.3)	45 (81.8)	
Former smoker	42 (19.4)	36 (22.2)	6 (10.9)	
Current smoker	42 (19.4)	38 (90.5)	4 (9.5)	
Smoking frequency^c^ (pack‐year) median [IQR] (*n* = 84)	26.6 [15.0–44.8]	28.5 [15.0–46.3]	23.0 [9.3–32.5]	0.312
Intensity of smoking^b^ *N* (%)				0.001
Nonsmoker	133 (61.3)	88 (54.3)	45 (81.8)	
Light smoker	26 (12)	22 (13.6)	4 (7.3)	
Heavy smoker	58 (26.7)	52 (23.1)	6 (10.9)	
Duration of smoking^c^ (years), median [IQR] (*n* = 75)	30.0 [20.0–40.0]	30.0 [20.0–40.0]	26.0 [10.0–37.5]	0.175

Abbreviations: EGFR, epidermal growth factor receptor; IQR, interquartile range.

^a^ Independent *t* test.

^b^ *χ* ^2^ test.

^c^ Mann–Whitney *U* test.

* *p* value less than 0.05 is considered a significance.

The univariate logistic regression analysis showed that gender, smoking status, and smoking intensity were significantly correlated with EGFR mutations. The female gender increases the risk of EGFR mutations 2.5 times (odds ratio [OR]) more than the male gender (CI: 1.337–4.670, *p*: 0.004). Current smokers were five times less prone to EGFR mutations than nonsmokers (CI: 0.069–0.613, *p*: 0.005). Heavy smokers were also five times less prone to EGFR mutations than non‐smokers (CI: 0.090–0.565, *p*: 0.001). However, the results from the multivariate logistic regression analysis, as summarized in Table 3, did not demonstrate any significant associations between the variables and EGFR mutations.

**Table 3:** Univariate and multivariate logistic regression analyses of various predictive factors for EGFR status in patients diagnosed with non‐small cell lung cancer.

Variables	Univariate analysis			Multivariate analysis		
	OR	95% CI	*p* Value	OR	95% CI	*p* Value
Age	1.001	0.997–1.025	0.953	1.004	0.976–1.032	0.784
Gender						
Male	1			1		
Female	2.498	1.337–4.670	0.004	2.070	0.922–4.649	0.078
Smoking status						
Nonsmoker	1			1		
Former smoker	0.326	0.128–0.831	0.019	0.410	0.472–8.514	0.189
Current smoker	0.206	0.069–0.613	0.005	0.360	0.095–1.369	0.134
Smoking intensity						
Nonsmoker	1			1		
Light smoker	0.356	0.116–1.094	0.071	2.005	0.472–8.514	0.346
Heavy smoker	0.226	0.090–0.565	0.001			
Duration of disease	1.021	0.994–1.049	0.125	1.014	0.985–1.043	0.358

Abbreviations: 95% CI, 95% confidence interval; EGFR, epidermal growth factor receptor; OR, odds ratio.

## 4 DISCUSSION

The present study evaluated the incidence of EGFR mutations among smokers and nonsmokers with lung adenocarcinomas. Previous surveys have established a connection between adenocarcinoma histology and the high prevalence of EGFR gene mutations.^16^, ^17^ Consistent with these findings, our study found that 25.3% of patients diagnosed with adenocarcinomas tested positive for EGFR mutations mostly the deletion in exon 19, which aligns with previously published articles.^7^ Furthermore, EGFR mutations were found to be more prevalent among females. Similarly, other studies have shown that the rates of positive EGFR mutations were higher among female patients with adenocarcinomas, particularly among nonsmokers.^14^, ^18^, ^19^ In contrast, Kosaka et al. suggested that while adenocarcinoma histological type and nonsmoking status were the independent predictors of positive EGFR mutations, the female gender itself was not. They reported that the incidence of EGFR mutations was not higher among premenopausal females and proposed that the disparity between males and females in terms of EGFR mutation frequency could be attributed to different smoking habits, as a larger proportion of females tended to be never smokers.^16^

A molecular epidemiological study has provided evidence of a link between specific environmental carcinogens, including those found in tobacco smoke, and particular gene mutations in cancer progression. The results showed that a significant proportion of EGFR‐positive patients were nonsmoker.^20^ Therefore, smoking behavior was found to play a considerable role in the frequency of EGFR mutations and the survival of patients with NSCLC.^21^ In the present research, a notable proportion of EGFR mutants were nonsmokers. In the patients with a positive smoking history, the median pack‐year of smoking was higher in the wild‐type EGFR group than in the mutant ones. However, the differences became statistically significant when the overall smoking intensity was categorized into three categories: nonsmokers, light smokers, and heavy smokers. Although EGFR mutations tended to be more common among nonsmokers, they were not exclusive to this group.^22^, ^23^ Another study assessed the correlation between EGFR mutations and tobacco use. The findings indicated that patients who had quit smoking more than 20 years ago exhibited a similar EGFR mutation rate to never smokers.^24^ Although the present study demonstrated that less exposure to cigarette smoking would increase the likelihood of positive EGFR mutations, former smokers were five times less prone to mutation compared to never‐smokers which were significant.

For years, platinum‐based systemic chemotherapy served as the standard treatment for patients with NSCLC, resulting in overall survival of fewer than 2 years.^25^, ^26^, ^27^ Following the identification of activating EGFR mutations in a specific subgroup of NSCLC patients, targeted therapies such as TKI have emerged as highly effective treatments.^7^, ^28^ The use of anti‐EGFR TKIs has greatly improved patients' quality of life and extended their overall survival by approximately 12 months.^28^ Nonetheless, not all EGFR mutations are sensitive to TKIs.^29^ Specifically, those with an exon 21 or an exon 19 deletion mutations are sensitive, while those with an exon 20 mutation do not respond favorably.^29^, ^30^ Hence, determining the tumor's genotype has become crucial in planning treatment regimes in NSCLC patients, as the EGFR mutation status can predict the response to TKI drugs.^31^

Estimation of the probability of EGFR mutations using common demographic information has been proposed as a valuable adjunct to laboratory testing especially in developing countries and regions without enough facility.^32^ Information on the epidemiology of EGFR mutations and smoking patterns in our region is limited. The single‐center study evaluated EGFR mutations in 103 Iranian NSCLC patients and showed 24% mutation frequency mostly point mutation on exon 21 and never smokers.^33^ Another study with 50 patients reported EGFR‐positive mutations in 28% of patients. Females, nonsmokers, and deletion in exon 19 were more common in the EGFR‐positive group.^34^ Mohammadi et al. also reported a 20% frequency of EGFR mutations among patients with lung adenocarcinoma mostly on exon 19 and females.^35^

International guidelines recommend testing for EGFR mutations in all advanced nonsquamous NSCLC patients regardless of their gender, age, and smoking status. Additionally, it is recommended for all nonsmoker patients with advanced squamous NSCLC who are under the age of 50.^36^ However, in different regions of our developing country, due to the need to optimize patient services, this test is currently limited to nonsmokers and female patients with NSCLC. Nevertheless, our findings have highlighted a remarkable percentage of male patients and smokers among NSCLC cases. Consequently, smokers and male patients may also benefit from EGFR mutation testing and subsequent targeted therapies. Therefore, it is imperative to update the previous paradigm of EGFR mutation testing to middle‐aged, middle‐eastern, nonsmoker females should be updated to all NSCLC patients as major advancements in targeted therapy and patient survival among NSCLC patients harboring EGFR mutations have been achieved in recent years.^37^

This study conducted in our region was the first and had the largest sample size to evaluate the relationship between smoking patterns and EGFR mutations. While the potential effect of ethnicity should not be underestimated, it is important to note that the study sample consisted of a homogenous group of patients from southern Iran, which enabled a more accurate analysis. Furthermore, the DNA samples were analyzed by highly expert pathologists in well‐equipped and reliable laboratories.

Based on the findings of the present study, it is recommended that EGFR mutation testing be routinely performed for all patients diagnosed with NSCLC regardless of gender and smoking history considering that EGFR mutations were identified in a significant proportion (25.3%) of patients with lung adenocarcinoma and also it can still occur in nonsmokers and light smokers. This approach will ensure that patients who are eligible for targeted therapies based on their EGFR mutation status receive appropriate treatment options.

However, the study faced several limitations due to its retrospective nature. First, the small number of female patients could potentially affect the interpretation of the results. Second, although all the accessible data were collected from the laboratories and patients' records, information regarding cancer stage and grade was not available. Third, due to the poor health condition of some patients, as well as cases of mortality and changes in contact information, it was not possible to gather missing information from them. In addition to the aforementioned limitations, most of the patients had reduced their consumption of waterpipes (hookah) after their disease diagnosis. Consequently, accurate data on hookah usage status was not available.

## 5 CONCLUSION

In conclusion, consistent with numerous published studies, a strong association was observed between positive EGFR mutations, particularly in exon 19, and female gender as well as nonsmoker status. Considering the detection of positive EGFR mutations in smokers and the survival benefits associated with targeted therapies, it is recommended that routine mutation testing be performed for all NSCLC patients, regardless of their clinical and demographic characteristics. This study presented the largest EGFR mutation database in our region, focusing on smoking profiles. Given the limited accessibility of well‐equipped EGFR testing laboratories for a significant number of patients in developing countries, the findings of such epidemiological surveys can serve as valuable guidance in determining the optimal long‐term treatment strategy.

## AUTHOR CONTRIBUTIONS

**Seyedeh Yasamin Parvar**: Methodology; writing—original draft; writing—review and editing. **Alireza Rezvani**: Conceptualization; project administration; resources; supervision; validation. **Rezvan Ghaderpanah**: Data curation; formal analysis; writing—review and editing. **Mohammadhossein Hefzosseheh**: Data curation; Methodology; Writing—original draft. **Shakila Rafiei**: Data curation; writing—original draft. **Ahmad Monabati**: Conceptualization; supervision; validation.

## CONFLICT OF INTEREST STATEMENT

The authors declare no conflict of interest.

## ETHICS STATEMENT

The manuscript has been approved by all authors and has never been published or under consideration for publication elsewhere. We confirm that all figures and tables are original and created by authors. We guarantee that all authors listed on the title page have read the manuscript and attest to the validity and legitimacy of the data. We would also like to undertake that we have read the plagiarism policy and submitted the article with complete responsibility. This study was done in compliance with the Declaration of Helsinki and approved by the ethics committee of the university. All patients gave their informed consent before their inclusion in the study.

## TRANSPARENCY STATEMENT

The lead author Alireza Rezvani affirms that this manuscript is an honest, accurate, and transparent account of the study being reported; that no important aspects of the study have been omitted; and that any discrepancies from the study as planned (and, if relevant, registered) have been explained.

## Supporting information

Supplementary information.

## Data Availability

The data that support the findings of this study are available on request from the corresponding author. The data are not publicly available due to privacy or ethical restrictions.
