# Supplementary material for: The relation between epidermal growth factor receptor mutations profiles and smoking patterns in patients with lung adenocarcinoma: A cross‐sectional study
Source: Health Sci Rep. 2023 Jul 5;6(7):e1369. doi: 10.1002/hsr2.1369 (PMC10323165; doi:10.1002/hsr2.1369)
Supplement: Supplementary file 1 — Supplementary information. [file HSR2-6-e1369-s001.docx]

**Supplementary Table 1.** EGFR Mutation subtypes in this study

| Mutations | Mutations subtype |
| --- | --- |
| Exon19 | Deletion/insertion in exon 19; c.2235_2249del (p. ∆E746-A750 deL); COSM13243 |
| Exon 21 | Point mutation in exon 21; c.2573T>G (L858R); COSM6224 |
| Exon 18 | The missense mutation in exon 18, c.2155G>A (p. Gly719Ser) |
| Exon 20 | Insertion in exon 20 |
